# The Health-Promoting Potential of Wafers Enriched with Almond Peel

**DOI:** 10.3390/molecules31010129

**Published:** 2025-12-30

**Authors:** Urszula Szymanowska, Monika Karaś, Ivo Oliveira, Sílvia Afonso, Barbara Chilczuk, Katarzyna Lisiecka

**Affiliations:** 1 Department of Biochemistry and Food Chemistry, Faculty of Food Sciences and Biotechnology, University of Life Sciences in Lublin, 8 Skromna Str., 20-704 Lublin, Poland; 2 Centre for the Research and Technology of Agroenvironmental and Biological Sciences, CITAB, Inov4Agro, Universidadede Trás-os-Montes e Alto Douro, UTAD, Quinta de Prados, 5000-801 Vila Real, Portugal; 3 Department of Chemistry, Faculty of Food Sciences and Biotechnology, University of Life Sciences in Lublin, 15 Akademicka Str., 20-950 Lublin, Poland

**Keywords:** almond peel, wafers, functional food, phenolic compounds, antioxidant activity, enzyme inhibition, antiproliferative potential

## Abstract

This study aimed to evaluate the health-promoting potential of wafers enriched with almond peel as a natural source of bioactive compounds. Wafers were prepared with different concentrations of almond peel (1%, 2%, 5%, and 10%) and analyzed to determine their phenolic content, antioxidant capacity, enzyme inhibition, anticancer properties, and sensory properties. Three types of samples were examined: buffer extracts (PBS), ethanol extracts (EtOH), and samples obtained after in vitro digestion (TRW). Antioxidant properties were assessed using ABTS^+•^ and DPPH^•^ assays, as well as Fe^2+^ chelation and reducing power tests. Enzyme inhibitory activities against LOX, COX, ACE, and lipase, and antiproliferative potential of hydrolysates toward AGS and HT-29 cell lines were also determined. The highest levels of total phenolic, flavonoids, and phenolic acids were found in digested samples of wafers with 10% almond peel addition (W10), reaching 2.243 mg/g, 6.153 µg/g, and 0.554 mg/g, respectively, while PBS extracts of control wafers (WK) showed the lowest values (0.159 mg/g, 0.146 µg/g, and 0.316 mg/g, respectively). The digested W10 samples showed the strongest antioxidant and enzyme inhibitory activities. The wafer hydrolysates caused only a modest reduction in HT-29 cell viability, and this effect was observed exclusively at the higher concentrations tested. The results confirm that almond peel enhances the health-promoting properties of wafers.

## 1. Introduction

In line with the global zero-waste trend, increasing attention is being directed toward minimizing waste and valorizing byproducts generated during food processing. Almond processing yields considerable quantities of byproducts, which are often discarded [1].

Waste generated during almond processing includes: almond shells (over 6 million tons), husks (0.8–1.7 million tons), blanching water, and almond peels [2,3]. During almond processing, different kinds of wastes are produced: green hull—52%, shell—33% and peel after skinning the kernel—15% of the total fresh weight [3,4,5,6].

The growing interest in the utilization of byproducts is driven, among other factors, by the presence of numerous bioactive compounds and their associated health-promoting properties.

Recent reviews on the valorization of nut byproducts, including almonds, highlight their potential as sources of functional ingredients within sustainable food systems [3,5,7,8].

The human body is continuously exposed to the harmful effects of reactive oxygen species (ROS). These free radicals possess strong oxidizing properties, which can contribute to the development of various diseases, including diabetes, asthma, and atherosclerosis. Antioxidant compounds mitigate the detrimental effects of free radicals and play a crucial role in maintaining physiological homeostasis. Phenolic compounds are among these key protective molecules [9].

Almond peel comprises approximately 4% of the entire fruit, but a significant portion of the phenolic compounds is found in it. Phenolic compounds found in almond peel include flavanols and flavonoid glycosides. Identified flavanols include catechin and epicatechin. Isorhamnetin-3-O-rutinoside and kaempferol-3-O-rutinoside are the main flavonol glycosides present in almond peel. Isorhamnetin, present as either a 3-O-glucoside or a 3-O-rutinoside, accounts for approximately 70% of the flavonoid content in the skin. Eleven of the nineteen phenolic compounds identified in almonds were found exclusively in the skin [3,10,11,12].

Enzyme inhibitory assays are widely used to evaluate the potential health-promoting properties of bioactive compounds in food and plant-based materials [13,14,15]. Inhibition of specific enzymes can provide insight into the anti-inflammatory, antihypertensive, and anti-obesity effects of the tested samples. Lipoxygenase (LOX) and cyclooxygenase-2 (COX-2) are key enzymes involved in the inflammatory process, and their inhibition is associated with a reduction in the production of pro-inflammatory mediators. Angiotensin I-converting enzyme (ACE) plays a central role in blood pressure regulation, and ACE inhibitors are important in managing hypertension [16]. Pancreatic lipase is critical for dietary fat digestion, and its inhibition can reduce lipid absorption, contributing to potential anti-obesity effects [17,18].

The assessment of enzyme inhibitory activity allows for the identification of bioactive compounds that may modulate these physiological pathways, providing a scientific basis for the development of functional foods or nutraceuticals with health-promoting properties.

Almond waste products are increasingly being used to enrich existing food products and can be a raw material for the production of high-value-added food [4,19]. Recent research demonstrated that almond peels have a range of bioactive properties, including antibacterial, anti-inflammatory, prebiotic, and anticancer activities, which are due to the polyphenolic compounds present in them [3]. Moreover, current reviews emphasize the importance of applying standardized in vitro digestion models to assess the bioaccessibility and stability of bioactive compounds in processed food matrices [20]. This topic remains insufficiently researched in the case of bakery products enriched with almond peel.

Consuming foods rich in polyphenols reduces the risk of chronic diseases in the future. Nowadays, food producers are increasingly paying attention not only to the organoleptic properties of their products but also to their health-promoting properties, including enriching their food with bioactive ingredients [19]. Almond peel is currently underutilized in the food industry, yet it may represent a promising ingredient for functional foods. An example of the use of almond peels as a byproduct is their use in wafer production [21].

Despite growing interest in the utilization of almond byproducts, no previous study has comprehensively examined almond peel-enriched wafers using an integrated approach that combines chemical analysis, enzyme inhibitory activity, cytotoxicity assessment, and a standardized in vitro digestion model. Therefore, this study provides novel insight into how almond peel fortification influences the bioactive profile and biological properties of wafers before and after simulated gastrointestinal digestion.

The study aimed to investigate the effect of fortifying wafers with almond peels on the content of bioactive compounds and their health-promoting properties. Particular emphasis was placed on the potential activity of these wafers after simulated digestion. Their consumer quality was also examined.

## 2. Results

### 2.1. The Consumer Evaluation

**Figure 1:**
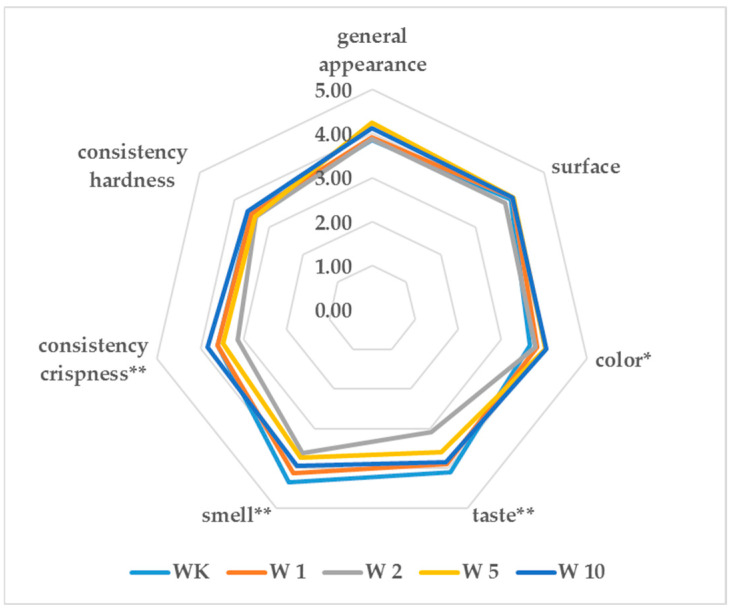
The consumer evaluation of control wafers (WK) and wafers enriched with almond peel at 1%, 2%, 5%, and 10% (W1, W2, W5, and W10, respectively). Significant differences between samples were indicated by asterisks (* *p* < 0.05, ** *p* < 0.01, Tukey’s test).

Wafers prepared with various concentrations of almond peel added and control wafers that did not contain almond peel were subjected to consumer evaluation. The results of the assessment are presented in Figure 1. The color, smell, and taste of wafers are some of the most significant characteristics, as they have a decisive impact on the attractiveness of the product. When analyzing the graph in Figure 1, it can be observed that the consumer evaluation of the wafers (represented by the line connecting the midpoints of the sample descriptors) followed the trend corresponding to the percentage of almond peel added to their formulation. The samples differed statistically in the descriptors of taste, smell, crispness, and color. For the remaining descriptors, no statistically significant differences were found. The effect of almond peel flour addition on the surface, hardness, and overall appearance of the wafers was relatively small, suggesting that it would not negatively influence the overall quality of the product. Overall appearance of the wafers was rated between 3.85 and 4.25. The wafers with 10% almond peel received the highest rating in terms of color, while the control variant received the lowest rating.

### 2.2. Bioactive Compound Content

**Figure 2:**
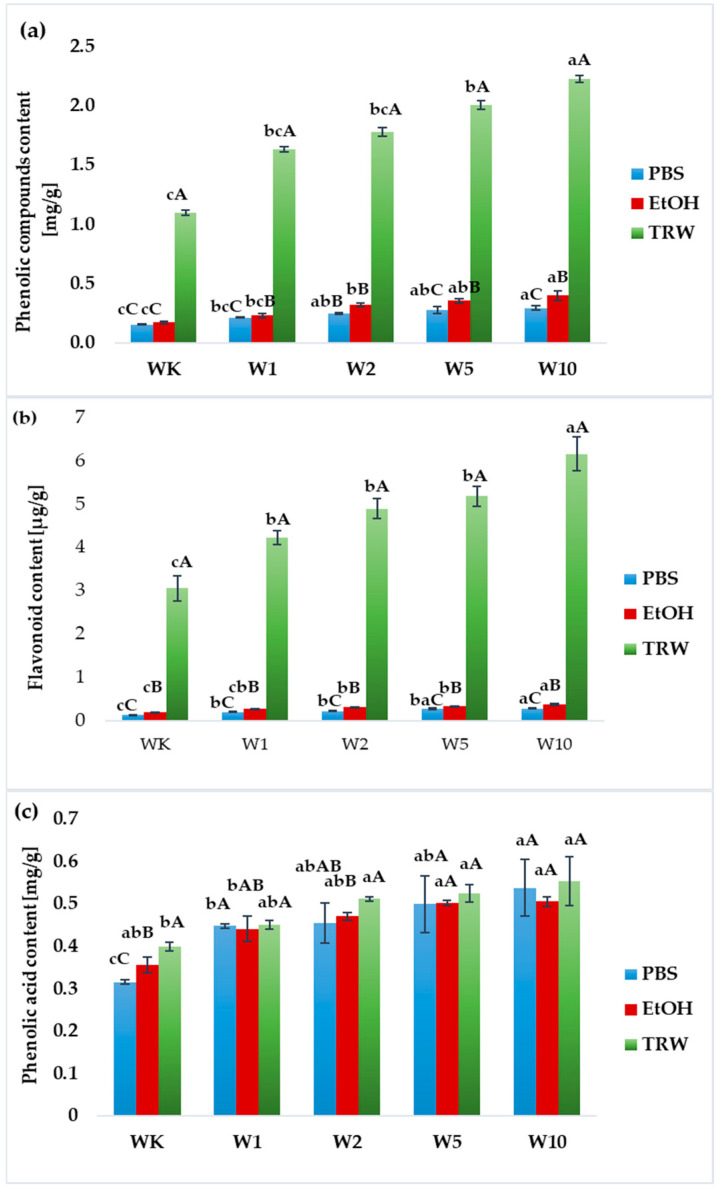
Total phenolic compounds (**a**), flavonoids (**b**), phenolic acids (**c**) in PBS, EtOH extracts, and in vitro digestion samples (TRW). WK—control wafers; W1–W10—wafers with almond peel addition (from 1 to 10% flour substitution, respectively). All values are mean ± standard deviation for triplicate experiments. Different lowercase letters indicate significant differences between wafers WK–W10 (*p* < 0.05), whereas different uppercase letters indicate significant differences between samples depending on the extraction system (ethanol, PBS) and digestion (*p* < 0.05).

When comparing the results obtained in three different types of samples (PBS extracts, ethanol extracts, and after in vitro digestion), the highest content of total phenolic compounds and flavonoids was found in the samples after digestion, and the lowest in the PBS extracts. All W1–W10 extracts exhibited higher contents of phenolic compounds, flavonoids, and phenolic acids compared to the control sample (WK) (Figure 2a–c). The total phenolic content (Figure 2a) ranged from 0.159 mg/g in the control sample to 2.243 mg/g in the sample containing 10% almond peels. The flavonoid content varied from 0.146 to 6.153 µg/g (Figure 2b), while the phenolic acid content ranged from 0.316 to 0.554 µg/g (Figure 2c). For the results obtained from the determination of phenolic acid content, no statistically significant differences were observed between extracts and hydrolysates within the same type of wafers; however, explicit differences were noted between the control wafers (WK) and those enriched with 10% almond peel (W10).

The extracts and hydrolysates of control wafers (WK) and wafers enriched with 10% almond peel (W10) were subjected to LC-QTOF-MS analysis to identify bioactive compounds and compare their profiles (Table 1). The analysis revealed the presence of several classes of compounds, including phenolic acids (caffeic acid, ferulic acid, p-coumaric acid), flavonoids (apigenin derivatives, catechin, epicatechin, kaempferol, and isorhamnetin glycosides), amino acids (tryptophan, valine, phenylalanine, tyrosine, arginine, and isoleucine), as well as vitamins (B2, B3, and B5), see Table 2. In addition, compounds characteristic of almond peels, such as benzaldehyde and mandelic acid, were detected exclusively in W10 samples. Overall, the almond peel-enriched wafers (W10) exhibited a richer and more diverse polyphenolic profile compared to control wafers (WK), which may explain their higher antioxidant potential and possible health-promoting properties. The extraction solvent strongly influenced the compound profile. Ethanolic extracts and extended extraction time favored the recovery of flavonoids and polyphenols, whereas PBS extracts were richer in amino acids and selected vitamins. These results highlight the importance of solvent choice and extraction conditions in maximizing the recovery of bioactive compounds.

**Figure 3:**
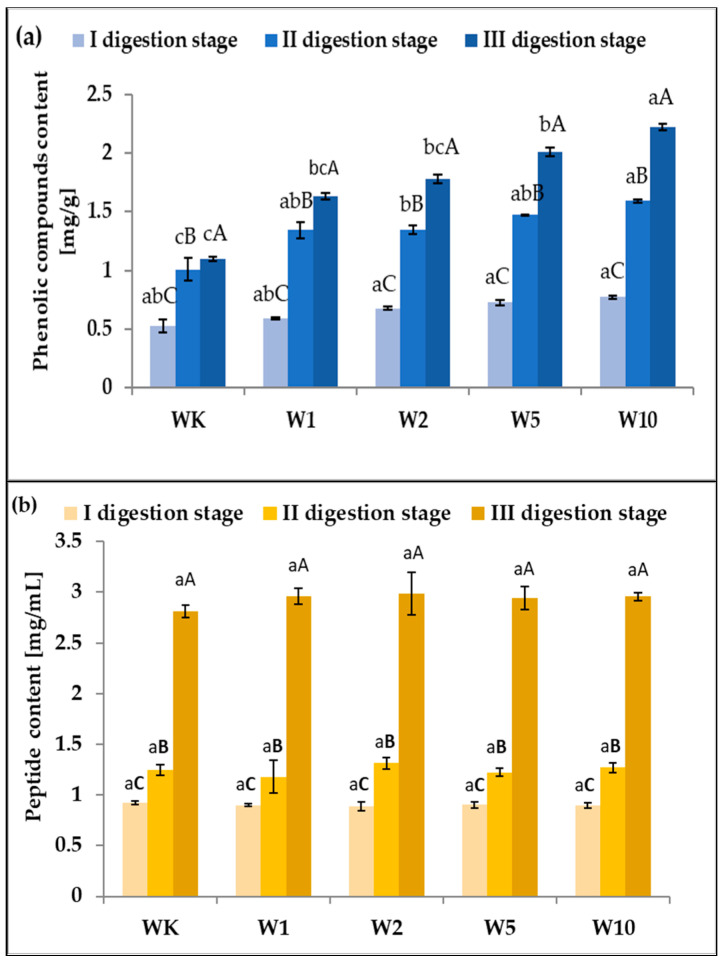
Phenolic compound (**a**) and peptide (**b**) content in samples during in vitro digestion of wafers enriched with almond peel. All values are mean ± standard deviation for triplicate experiments. Different lowercase letters indicate significant differences between wafers WK–W10 (*p* < 0.05), whereas different uppercase letters indicate significant differences between samples depending on the digestion stage (*p* < 0.05).

The release of polyphenols and peptides present in each type of wafer’s hydrolysates after simulated oral, gastric, plus duodenal digestion is reported in Figure 3.

The results of the presented study indicate that the presence of a food matrix had a significant effect on polyphenol bioaccessibility from almond peel. Polyphenol content increased after each digestion step. After simulated gastric digestion, approximately twice as many compounds were released in all wafer samples at low pH. However, after the third step of in vitro digestion, the greatest differences were observed for the wafers enriched with almond peel, especially at the highest concentration used.

Proteins derived from food are decomposed in the stomach by pepsin, and then further down the gastrointestinal tract by trypsin, chymotrypsin, and many other proteases and peptidases. The results obtained in this study confirm this, as an increase in peptide content is observed at each stage of protein digestion (Figure 3b). Following intestinal digestion, more than twice as many peptides are released as after gastric digestion. However, there are no statistically significant differences between the individual wafer variants, confirming that the main source of protein in the wafers was not almond husks but the matrix.

### 2.3. Antioxidant Properties

**Figure 4:**
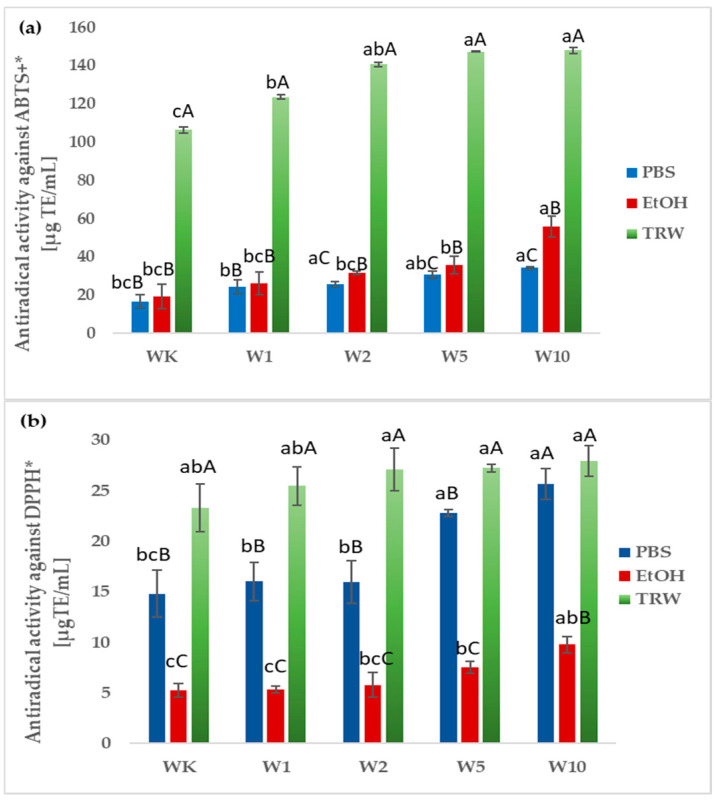
Antiradical activity against ABTS^+^* (**a**) and DPPH* (**b**) of PBS and ethanol extracts, and samples obtained after in vitro digestion (TRW). All values are mean ± standard deviation for triplicate experiments. Different lowercase letters indicate significant differences between wafers WK–W10 (*p* < 0.05), whereas different uppercase letters indicate significant differences between samples depending on the extraction system (ethanol, PBS) and digestion (*p* < 0.05).

To determine the antioxidant properties of samples, methods based on the determination of antiradical activity using ABTS and DPPH radicals, as well as on the assessment of the iron ion-chelating capacity and reducing potential, were used. This study showed that free radicals generated from ABTS and DPPH were scavenged by all samples (Figure 4). The best results were obtained for in vitro digested wafers (140.63–147.90 µg TE/mL) with no significant differences. Digested samples exhibited the highest radical scavenging capacity, followed by ethanol extracts, while PBS extracts showed the lowest activity. In PBS and ethanol extracts, the highest statistically significant ABTS radical scavenging activity was observed for sample W10 (34.16 and 55.78 µg TE/mL, respectively), while the lowest activity was found for WK (16.79 and 19.16 µg TE/mL, respectively). The results for WK and W1 did not differ significantly (Figure 4a). In ethanol extracts, the antioxidant activity against the ABTS radical increased proportionally with the rising concentration of almond peel addition. In the case of testing the ability to scavenge free radicals ABTS, it was noticed that, regardless of the extract used, this ability was positively correlated with the content of bioactive compounds, except for the ethanol extract, for which there was no significant correlation between the content of phenolic acids and the tested activity. While in the case of DPPH radical scavenging capacity, significant correlations were found between total polyphenol content, flavonoid content, phenolic acid content, and the tested activity in the extract after digestion and between this activity and flavonoid content in the PBS extract (correlation matrix available in the Supplementary Table S1).

**Figure 5:**
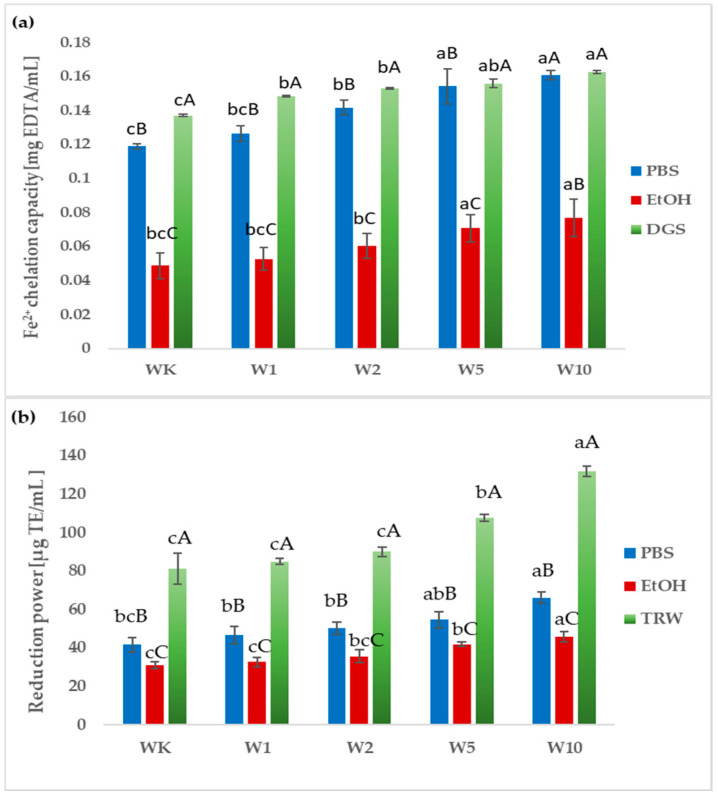
Fe^2+^ ion-chelating capacity (**a**) and reduction power (**b**) of PBS, EtOH extracts, and in vitro digestion samples (TRW). All values are mean ± standard deviation for triplicate experiments. Different lowercase letters indicate significant differences between wafers WK–W10 (*p* < 0.05), whereas different uppercase letters indicate significant differences between samples depending on the extraction system (ethanol, PBS) and digestion (*p* < 0.05).

In PBS extracts, the highest Fe^2+^-chelating activity was observed in W10 (0.160 mg EDTA/mL) and the lowest in WK (0.119 mg EDTA/mL), while W1 and W2 showed similar levels. However, no statistically significant differences were found between the samples W5 and W10 (Figure 5a). Similarly, ethanol extracts of WK–W10 wafers showed no significant differences in Fe^2+^-chelating capacity. For in vitro digested samples, Fe^2+^-chelating activity slightly increased with higher almond peel content, with the highest in W10 (0.163 mg EDTA/mL) and the lowest in WK (0.137 mg EDTA/mL). Overall, Fe^2+^-chelating capacity was similar between in vitro digested samples and PBS extracts, whereas ethanol extracts showed markedly lower activity (Figure 5a). Additionally, it was noted that in the case of all extracts, the ability to chelate metal ions was strongly positively correlated with the content of total phenolic compounds, flavonoids and phenolic acids (correlation matrix available in Table S1).

The reducing power of the analyzed samples, expressed as µg TE/mL, varied significantly depending on the extraction solvent and the addition level of almond peel (Figure 5b). Among all extracts, the TRW exhibited the highest reduction power across all samples (WK–W10), whereas the EtOH (ethanol) extracts showed the lowest values. The PBS extracts demonstrated intermediate activity. A gradual increase in reducing power was observed with higher almond peel enrichment, reaching the maximum value for the W10 sample (131.74 µg TE/mL). This indicates a dose-dependent enhancement of antioxidant capacity in wafers enriched with almond peel. In contrast, the control sample (WK) showed significantly lower reducing power compared to enriched samples. However, only in the case of PBS extracts a significant positive correlation was observed between all the tested bioactive compounds and the ability. In the case of the ethanol extract, no significant correlation was observed between the phenolic acid content and the tested activity. In the case of the extract after the digestion, a significant correlation was observed only between the flavonoid content and the reducing capacity (correlation matrix available in the Supplementary Table S1).

### 2.4. Inhibition of the Activity of Enzymes Involved in the Pathogenesis of Obesity, Hypertension, and Inflammation

The potential anti-hypertensive, anti-obesity, and anti-inflammatory properties of wafers enriched with almond peels were also evaluated. For this purpose, ACE, lipase, LOX, and COX-I inhibitory activity assays were carried out. Data are expressed as EC_50_ values, representing the concentration required to achieve 50% inhibition. The results obtained from PBS, EtOH, and TRW extracts are summarized in Table 3.

Both ACE and lipase inhibitory activities increased with the concentration of almond peels in the wafers. The strongest effects were observed in digested fractions (TRW), followed by ethanol extracts, while PBS extracts showed the weakest activity. The EC_50_ values of ACE inhibition ranged from 0.226 ± 0.027 to 9.680 ± 1.065. The lowest EC_50_, indicating the strongest inhibitory effect, was observed for the hydrolysate of the wafer with 10% almond peel additives (W10, 0.226 ± 0.027). A similar trend was observed for the lipase inhibition assay, where the addition of almond peel also led to a concentration-dependent improvement in inhibitory potential. For hydrolysates (TRW), the EC_50_ values ranged from 1.780 ± 0.178 (WK) to 0.232 ± 0.024 (W10), and were markedly lower compared to the values obtained for ethanol and PBS extracts. This study also analyzed the potential anti-inflammatory properties of wafers fortified with almond peels. For this purpose, the effect of the obtained extracts on the activity of enzymes involved in arachidonic acid metabolism (LOX and COX-2, which can lead to the formation of pro-inflammatory compounds) was examined. In both cases, buffer extracts did not demonstrate the ability to inhibit these enzymes at the concentrations used. However, for both ethanol extracts and those obtained after in vitro digestion, EC_50_ values were determined, indicating the extract concentration that inhibits enzyme activity by 50%. For the ethanol extracts, these values ranged from 2.639 ± 0.157 mg/mL for WK to 2.042 ± 0.397 mg/mL for W10 for COX-2 and from 2.655 ± 0.273 mg/mL for W1 to 2.042 ± 0.397 mg/mL for W10 for LOX, with the control wafers showing no inhibitory activity. Extracts after simulated digestion showed significantly lower EC_50_ values, meaning higher inhibition force. Wafers with 10% almond peel added demonstrated the highest LOX inhibition effectiveness. The remaining additive concentrations were too low. In turn, in the case of cyclooxygenase-2, the potential reduction in inflammation occurred already at the lowest concentration of the additive, although digested W10 wafers were the most effective. In the case of testing the ability to inhibit angiotensin-converting enzyme and lipase, a significant correlation was observed between the total content of polyphenolic compounds, flavonoids, and phenolic acids and the ability to inhibit these enzymes in all tested extracts. A similar relationship was also observed in the case of COX-2 inhibition (except for PBS, which showed no activity). In the case of LOX inhibition, statistically significant correlations were observed between the tested compounds and the ability to inhibit the enzyme only in the case of digested extracts (correlation matrix available in Supplementary Table S1).

### 2.5. Potential Antiproliferative Activity

Wafer samples obtained after simulated digestion were analyzed for their potential anticancer properties. Their effect on the viability of gastrointestinal cancer cells was examined.

**Figure 6:**
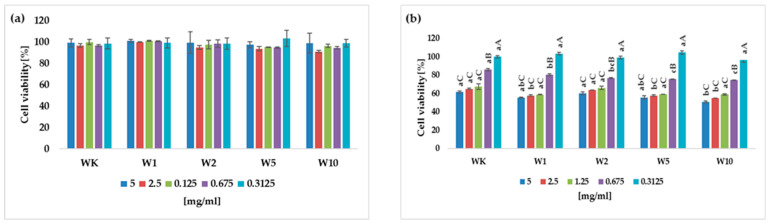
Antiproliferative activity of digest from wafers: (**a**) AGS—gastric adenocarcinoma, (**b**) HT-29—colorectal adenocarcinoma. WK—control wafers; W1–W10—wafers with almond peel addition. All values are mean ± standard deviation for triplicate experiments. Different capital letters indicate statistically significant differences within the same sample but between the different concentrations of digest (*p* < 0.05). Values denoted by different small letters indicate statistically significant differences between the different samples within the same concentration (*p* < 0.05).

No antiproliferative activity was observed in the gastric adenocarcinoma AGS line (Figure 6a). Neither the control digested wafers nor the almond peel-fortified wafers significantly affected the viability of this cancer cell. Even very high, non-physiological concentrations did not inhibit the growth of the cells.

In vitro digested wafer samples affected the viability of the HT-29 colorectal adenocarcinoma cell line. The higher concentrations used in the study significantly reduced cell viability. The relationship between sample concentration and antiproliferative activity is dose–response and nonlinear. A sigmoidal model is most likely in this case—the extract’s effect is modest at low concentrations, increases rapidly within a specific concentration range, and then reaches a plateau at very high concentrations (Figure 6b). It is worth noting, however, that concentration (0.625 mg/mL used in the study), which may at least partially reflect the physiological situation in the gastrointestinal tract, resulted in a decrease in cell viability of this line. At this near-physiological concentration, a greater statistically significant decrease in cell viability can also be observed with increasing almond peel addition to the wafers. Moreover, even at the lowest extract concentration used (0.3125 mg/mL), a decreasing trend in the viability of HT-29 cells was observed with increasing amounts of the additive, although the differences between samples were statistically insignificant.

## 3. Discussion

Sensory analysis is a critical tool in food science, as it provides insights into consumer perception and acceptance of products, which are essential for their market success. The sensory properties of color, aroma, taste, texture, and overall appearance directly influence the attractiveness and acceptability of food products. In the study by Oliveira et al. [21], the sensory evaluation of wafers was performed by a small trained panel of nine tasters (seven women and two men) from UTAD, specialized in such assessments. In contrast, our semi-consumer evaluation of the same wafers involved a larger group of 40 employees and students from the University of Life Sciences in Lublin. Despite the difference in panel size and experience, both evaluations demonstrated that the addition of almond peel significantly influenced key sensory attributes, including taste, aroma, consistency, crispness, and color. In both studies, wafers with higher almond peel content, particularly the 10% formulation, were preferred, while surface texture, hardness, and overall appearance were minimally affected, indicating that product acceptability was maintained. The highest color score observed in the 10% almond skin addition group (W10) can be attributed to a combination of factors. Almond skin is rich in naturally colored phenolic compounds, such as flavonoids and tannins, which can impart a darker hue to the wafer matrix. Additionally, thermal processing during wafer baking may induce Maillard reactions between reducing sugars and amino acids, generating melanoidins that contribute to browning [22,23]. The increased presence of these pigmented compounds correlates with the enhanced antioxidant capacity observed in W10.

In this study, we aimed to evaluate the effect of adding almond peel to wafers on the health-promoting potential of the enriched product.

Particular attention was given to both the technological aspects and the effectiveness of food enrichment. Almond peel, as a fiber-rich ingredient, can influence both the structural and nutritional properties of wafers. High levels of fiber may dilute gluten and disrupt the protein matrix, limiting structure formation and causing shrinkage or deformation, particularly in thin, low-viscosity wafer batters [24,25,26]. These effects are consistent with Verbeke et al. (2024), who reported that substituting flour with fiber at 1%, 5%, or 10% increased water absorption and affected dough development, stability, and extensibility [27]. Negative effects were minimal at low fiber levels (up to 5%), but significant variability and structural challenges appeared at 10%, supporting this value as a practical technological limit for fiber enrichment in wheat-based products [27]. In wafers, these effects can be amplified, justifying the selection of up to 10% almond peel addition while still enhancing the product’s health-promoting potential.

Special attention was also given to the effectiveness of food enrichment in terms of the potential bioavailability of phenolic compounds and peptides. Analysis of total phenolic content revealed that the addition of almond peel significantly increased the levels of these compounds in both extracts and hydrolysates. Almond peel is rich in polyphenolic compounds, including flavonols, flavanols, and phenolic acids such as catechin, epicatechin, and kaempferol derivatives [6,12]. In contrast, the phenolic content of the control wafers originated solely from the flour used.

The present study demonstrated that the incorporation of almond peels into wafers significantly increased the content of phenolic compounds, flavonoids, and phenolic acids compared to the control samples. Similar trends have been reported by Oliveira et al. [21], although the absolute values differed due to variations in extraction solvents and analytical standards. In our study, the hydrolysates of wafers containing 10% almond peels exhibited markedly higher phenolic content (3.243 mg/g) compared to the control (0.159 mg/g), while flavonoid levels also showed a substantial increase, particularly after digestion (up to 6.153 µg/g). These findings suggest that almond peel not only enriches the phenolic profile of wafers but also enhances the bioaccessibility of these compounds during digestion, which may improve their potential biological activity. Similar effects of incorporating alternative ingredients on phenolic content and bioaccessibility in bakery products have also been described in the literature [28,29,30,31].

The higher flavonoid content determined by the Lamaison method after simulated digestion compared to ethanol extracts can be attributed to the principle of this assay. The aluminum chloride complexation method detects a broad group of flavonoids capable of forming stable complexes with Al^3+^. During digestion, glycosidic bonds may be hydrolyzed and flavonoids released from the food matrix, which increases the amount of reactive aglycones available for detection [32,33].

By contrast, MS analysis quantifies specific intact flavonoid structures. Under gastrointestinal conditions, some of these molecules undergo degradation or transformation into smaller phenolic acids that are no longer identified as flavonoids in MS. Therefore, ethanol extracts show higher concentrations of native flavonoids by MS, while the Lamaison method reflects the overall increase in reactive forms after digestion [34].

Based on LC-QTOF-MS analyses, the bioactive compounds present in the investigated material were identified.

The presence of phenolic acids and flavonoids may explain the high antioxidant activity and potential antihypertensive properties of wafers enriched with almond peels. Literature reports particularly emphasize the role of ferulic acid (FA) and p-coumaric acid (p-CA). Ferulic acid exhibits strong free radical scavenging activity due to its ability to donate hydrogen atoms and electrons. Its antihypertensive effect has been associated with angiotensin-converting enzyme (ACE) inhibition, vasorelaxation, and attenuation of metabolic disturbances, as confirmed in in vivo studies [35,36].

*p*-Coumaric acid acts as an effective free radical scavenger and inhibitor of radical generation, reducing lipid peroxidation and enhancing antioxidant defense mechanisms. Its antihypertensive effect is related to the modulation of the NO/cGMP signaling pathway, which promotes vascular relaxation [37,38,39]. In comparison, mandelic acid exhibits considerably weaker antioxidant and antihypertensive activities [40].

Among the identified flavonoids, kaempferol, catechin, epicatechin, and isorhamnetin (3-O-glucoside) demonstrated strong antioxidant and cardioprotective properties. Kaempferol functions as a free radical scavenger, protects antioxidant enzymes, and, in animal models, reduces blood pressure through ACE inhibition, modulation of the Ang II/AT1/NOX4 pathway, and induction of vascular relaxation [41]. Catechin and epicatechin neutralize free radicals, improve endothelial function by increasing nitric oxide (NO) bioavailability, reduce oxidative stress, and inhibit renin activity, thereby contributing to blood pressure reduction [42,43,44]. Isorhamnetin and its derivatives demonstrate ROS-scavenging capacity while their antihypertensive effects are associated with ACE inhibition and blockade of the PI3K/AKT signaling pathway, preventing cardiac hypertrophy and fibrosis [45].

Amino acids such as L-arginine and L-tryptophan may also play a significant role in the observed biological effects. Both compounds combine antihypertensive and antioxidant activities. Their mechanisms of action include enhanced nitric oxide synthesis, inhibition of vasoconstrictive enzymes (ACE, ECE, and renin), and reduction in oxidative stress [46,47,48]. Therefore, they may represent important factors supporting the prevention and management of hypertension and cardiovascular diseases.

Simulated digestion enhances the bioaccessibility and release of polyphenols and peptides or amino acids from the matrix, which likely contributes to the observed increase in ACE and lipase inhibition.

Several studies have reported on the effects of a food matrix in a simulated gastrointestinal environment on the release of bioactive compounds. The data presented by Mandalari et al. (2016) [20] demonstrated that the bioaccessibility of polyphenols from almond skin peel occurs in both gastric and intestinal environments and is significantly affected by the type of food matrix. A high release of bioactive compounds was observed from almond skin peel digested in water. However, a considerable loss of polyphenols was detected during the duodenal phase compared to the gastric environment, particularly in home-made biscuits and crisp-bread matrices. These findings support the current results, where simulated digestion (TRW) enhanced the release of bioactive compounds, leading to lower IC_50_ values and stronger ACE and lipase inhibition. The influence of the food matrix on polyphenol availability may explain some of the differences observed between PBS, ethanol, and digested extracts in this study. In a study of Bouayed et al. (2011) [49] on polyphenols bioaccessibility from apples indicated the release was mainly achieved during the gastric phase (65% of phenolics and flavonoids), with a slight increase (<10%) during intestinal digestion.

The present study demonstrated that the addition of almond peel to wafer formulations enhanced their bioactive potential, as reflected by the inhibition of ACE and pancreatic lipase. The inhibitory effect was dependent on the percentage of almond peel incorporated into the wafers, although in some cases, differences between intermediate concentrations were not statistically significant. Importantly, the simulated digestion distinctly enhanced the inhibitory activity, resulting in EC_50_ values approximately seven-fold lower than those observed in PBS and ethanol extracts. This observation suggests that gastrointestinal conditions may release or generate bioactive peptides and phenolic compounds with stronger enzyme-inhibitory properties [50].

ACE inhibition is a well-established therapeutic strategy for the management of hypertension, as ACE catalyzes the formation of angiotensin II, a potent vasoconstrictor. In the present study, wafers enriched with almond peel exhibited notably higher ACE inhibitory activity than the control wafers, with the 10% almond peel sample (W10) showing an EC_50_ of 0.226 mg/mL. By comparison, Jakubczyk et al. (2021) reported that hydrolysates from cookies enriched with 1% St. John’s wort after in vitro digestion showed increased ACE inhibitory activity (EC_50_ 1.24 mg/mL), although this effect was not statistically significant [51]. Similarly, Szymanowska et al. [52] raspberry/apple pomace (BP30) produced the greatest reduction in ACE activity (EC_50_ 1.16 mg/mL), but this was also not statistically significant. These results indicate that almond peel incorporation can substantially enhance ACE inhibitory potential, particularly after digestion, and may be more effective than other functional ingredient additions reported in previous studies.

This phenomenon aligns with existing literature, which highlights the role of in vitro gastrointestinal digestion in liberating bioactive peptides from food proteins. For instance, plant-derived ACE-inhibitory peptides are known to remain encrypted within precursor proteins and are released through enzymatic hydrolysis or digestion processes, increasing their bioactivity post-digestion [53]. Such findings support the hypothesis that almond peel proteins may similarly yield potent peptide inhibitors upon exposure to digestive enzymes.

In our study, we analyzed the potential anti-inflammatory properties of wafers fortified with almond peel. Buffered extracts did not demonstrate any inhibitory effects on enzymes involved in the pathogenesis of inflammation. However, extracts obtained after simulated digestion more effectively inhibited lipoxygenase and cyclooxygenase 2 activity compared to ethanol extracts. For LOX, EC_50_ values were 8–10 times lower for digested extracts, suggesting the effective release of compounds with anti-inflammatory potential from the food matrix. No simple linear relationship was found between the concentration of the additive (almond peel) and the ability to inhibit lipoxygenase activity. Extracts obtained from almond peels constitute a rich, heterogeneous mixture of various compounds, including polyphenols, peptides, and melanoid pigments formed through the Maillard reaction. Different components of this mixture may differ in their affinity for the enzyme and their mechanism of action. Therefore, the degree of inhibition is the result of the actions of all extract components, not their sum. The components of the mixture may interact, reinforcing or limiting the effectiveness of inhibition. Therefore, the possibility of interactions, both synergism and antagonism, may explain this nonlinear dose–response relationship [54].

Several research groups have previously reported the individual contents of the major active compounds in almond peel [3,6]. However, investigations into the possible interactions among the main constituents (lipids, polyphenols, and tocopherols) that coexist in these foods are still necessary.

Our extracts are multi-component mixtures of diverse nature, containing the main active substances and accompanying compounds (co-effectors) that have various effects on the components of the plant extract. They can alter the physicochemical properties of the main active substances, thus influencing their potential biological activity. Consequently, co-effectors can also alter the intensity of the active compounds’ effects. Co-effectors constitute the extract matrix, and their composition and properties depend closely on the extraction method and solvent used.

More research is needed to accurately determine the mechanisms of action and to analyze the type of interactions between individual antioxidants (synergism, antagonism, or no interactions).

For COX-2, the inhibition efficacy increased with increasing amounts of almond peel. For sample W10, the EC_50_ value was four times lower after digestion than for the ethanol extract. Polyphenols are able to affect inflammation processes through several mechanisms, also related to their antioxidative and radical scavenger properties [55]. Lauro et al. (2020) evaluated the radical scavenging activity and the pharmacological potential of almond peel extract in an in vitro model of intestinal inflammation, in intestinal epithelial cells [8]. Almond peel extract at concentrations of 10 and 50 µM inhibited the expression of TNF alpha, COX-2, and iNOS, which means it can be used in inflammatory diseases. Similarly, Mandalari et al. (2011) showed that natural almond peel (NS) powder reduced NF-κB and p-JNK activation, the pro-inflammatory cytokines release in a mouse model of inflammatory bowel disease [56].

The anti-inflammatory effects are closely linked to the antioxidant properties of polyphenols, including their ability to scavenge free radicals, reduce pro-oxidant species, and chelate metal ions. By mitigating oxidative stress, polyphenols can prevent the activation of redox-sensitive signaling pathways that promote inflammation, suggesting that their antioxidant activities play a key role in modulating inflammatory responses.

The antioxidant properties observed in the analyzed samples indicate their capacity to scavenge free radicals, reduce pro-oxidant compounds, and limit metal-catalyzed oxidation.

The increase in antioxidant activity detected after in vitro digestion appears to result not only from the enhanced release of phenolic compounds from the food matrix but also from chemical transformations occurring under gastrointestinal conditions [57]. The intestinal environment promotes hydrolysis of phenolic glycosides to their respective aglycones, which generally exhibit higher redox potential and improved electron-donating ability [58]. Moreover, the combined effects of pH shifts, digestive enzymes, and the presence of free amino acids and reducing sugars may facilitate the formation of Maillard products, several of which are known to display measurable antioxidant activity [59]. Additionally, interactions between liberated phenolics and reactive carbonyl species formed during digestion can yield phenolic–carbonyl adducts with enhanced radical-scavenging capacity. Collectively, these processes suggest that the post-digestive antioxidant response reflects both the bioaccessibility of native polyphenols and their biotransformation into structurally modified compounds with potentially higher functional activity [23].

The enhanced antioxidant capacity in wafers with 10% almond skin is likely due to the synergistic effect of phenolic compounds and Maillard reaction products, with phenolics both contributing natural pigmentation and acting as redox-active compounds that reinforce the bioactive properties of the final product. The increased presence of these pigmented compounds correlates with the enhanced antioxidant capacity observed in W10, as measured by reducing power and other assays. This suggests that the same polyphenols responsible for color intensity also act as effective electron donors, reinforcing the link between visual appearance and functional bioactivity.

Although skin represents only about 4.0% of the total almond, it contains about 60.0–80.0% of the total phenolic compounds existing in the nut. Phenolic compounds and iron form complexes that can vary depending on the chemical structure of the phenolic com-pound, with some being stronger chelators than others [60].

In particular, their ability to bind iron ions is attributed to the presence of catechol and gallate groups. Polyphenols, such as caffeic acid, gallic acid, and chlorogenic acid, exhibit a strong role in iron complexation due to the presence of a carboxylate group. Protein hydrolysates, which are a source of peptides, can also bind metal ions such as iron or copper [61]. Chelating activity depends on conditions such as environmental pH and the presence of other compounds, such as phytates and oxalates, which are present in almond peel and also strongly bind iron ions, forming insoluble complexes. They may compete with polyphenols for iron ions or dominate the binding process, masking the differences resulting from the polyphenol content itself [62]. Phytates are usually found in high quantities in almond peel (0.35–9.42%). The binding of non-heme iron by phytates can be significant, and can reach up to 50–65%. Phytic acid is the only element that, when combined with six coordination sites of iron, forms a completely inactive chelate, thus inhibiting the production of hydroxyl radicals and the oxidation of iron from Fe^2+^ to Fe^3+^ [63]. Therefore, the extracts’ ability to bind iron ions is a function of all the extract components, which may interact with each other. In this study, whole extracts were analyzed, so all components present may be responsible for their properties, including iron ion chelating. To determine the chelating capacity of the polyphenols themselves, it would be necessary to purify the extract and isolate the phenolic fractions, for example, on Sep-Pak^®^ C18 columns. Further analysis are needed to determine the exact mechanism, which requires specific biochemical tests.

To date, there are few scientific reports on the anticancer properties of almond peels, although many authors suggest such activity could result from the presence of biologically active compounds present in this raw material.There are even fewer studies examining the potential health-promoting properties, including anticancer properties, of products fortified with almond peels.

Picerno et al. (2023) found that aqueous almond peel extract does not imply cell viability reduction in A375, A549 cancer cell lines [64]. The authors’ conclusion that the extract obtained is safe compared to the healthy HaCaT cell line is crucial [64]. This study also demonstrated that the effect depends on the type of cancer. Not all cell lines are sensitive to the extracts we obtained. It should be noted that our studies used samples after in vitro digestion, which may indicate that the action of digestive enzymes and variable pH conditions may contribute to the release of compounds with potential biological activity from the food matrix [65]. However, biologically active compounds released from the food matrix must be absorbed in the gastrointestinal tract and transported to their target site of action.Therefore, it is difficult to estimate the probability of their delivery to gastric epithelial cells.This makes it difficult to assess their potential effects on distant tissues, such as gastric cells.The results of limiting the viability of colon cancer cells seem promising.Even if bioactive compounds are not absorbed, they reach the colon, where they can still exert their activity.The key conclusion is that even if bioactive compounds have poor systemic absorption, their activity in the colon is a critical factor for potential health benefits [66].

Analysis of cell viability in response to components of the analyzed extracts is only a preliminary study. The sensitivity of different cancer cell lines to phenolic compounds is highly variable and depends on the specific phenolic compound, the type of cancer, and specific cell signaling pathways. Phenolic compounds often exhibit selective antiproliferative effects on cancer cells while minimally affecting healthy, normal cells [67]. Their activity results from a variety of mechanisms, including the induction of apoptosis, cell cycle arrest, inhibition of angiogenesis, and modulation of signaling pathways, such as those related to oxidative stress. The sensitivity of different cell lines to oxidative stress varies and depends on their specific characteristics, such as metabolic requirements, mitochondrial efficiency, and antioxidant systems; thus, HT-29 cells could be more sensitive than AGS. The HT-29 cell line is extensively used in toxicology research, and Khodavirdipour et al. [68] a study explored the toxicity potential of a plant *Syzygium cumini* ethanolic extract on the HT-29 cell line. The results showed significant suppression of HT-29 cell growth, indicating the promising anticancer properties of the extract. Biological activity depends largely on bioavailability, which is determined by the compound’s chemical structure and metabolism in the body (e.g., intestinal absorption).

Extracts after in vitro digestion of wafers enriched with almond peel showed the presence of piceatannol 3-O-glucoside, which can inhibit the activity of matrix metalloproteinase (MMP)-9, which damages the extracellular matrix and promotes tumor growth [69]. This compound inhibits growth and induces apoptosis in many human cancer cell lines, including colon cancer, leading to decreased cell proliferation and cell death [70]. This topic requires deeper exploration and further in-depth analysis, including the mechanisms of antiproliferative action.

## 4. Materials and Methods

### 4.1. Chemicals

In this study, the following chemical reagents were employed: α-amylase, ABTS (2,2′-azobis(3-ethylbenzothiazoline-6-sulphonate) diammonium salt, acetonitrile HPLC grade, AlCl_3_ × 6H_2_O (aluminum chloride hexahydrate), bile salts, and caffeic acid (Sigma-Aldrich, Poznań, Poland). COX-2 colorimetric inhibitor screening assay kit was purchased from Cayman Chemical (Ann Arbor, MI, USA). DMEM (Dubelco’s modified Eagle’s medium) (Sigma-Aldrich, Poznań, Poland). 98% ethyl alcohol, ferricyanide K_4_[Fe(CN)_6_], ferrozine 3-(2-pyridyl)-5,6-bis-(4-phenyl-sulfonic acid)-1,2,4-triazone), FBS (fetal bovine serum), Folin–Ciocalteau reagent, formic acid, gallic acid, iron (II) chloride, linoleic acid, Na_2_MoO_4_ (sodium molybdate), NaNO_2_ (sodium nitrite), pancreatin, and PBS (buffered saline) were obtained from Chempur (Piekary Śląskie, Poland). Pepsin, Pen Strep, was purchased from Sigma-Aldrich (Poznań, Poland), and picrylsulfonic acid, phthaldialdehyde, p-nitrophenyl acetate (pNPA), quercetin, soybean lipoxygenase, TCA (trichloroacetic acid), Tween-20, and WST 1 cell proliferation reagent were obtained from Abcam (Cambridge, UK).

### 4.2. Preparation of Almond Peels

Almond hulls were removed, and shells were cracked to separate the kernels. Kernels were blanched in boiling water (95 °C, 3 min), and skins were manually peeled. Skins were oven-dried at 95–98 °C for 10 h to constant weight, vacuum-packed, and stored at room temperature [21].

### 4.3. Wafers Preparation

Wafers were prepared as described previously [21]. Briefly, sugar (130 g), eggs (4), melted and cooled butter (175 g), baking powder (1 tsp), and water (250 mL) were combined and mixed with a domestic hand mixer (Braun GmbH, Kronberg Taunus, Germany). The mixture was divided into five portions, with 50 g of flour replaced by almond peel at levels of 0% (WK as control), 1%, 2%, 5%, and 10% (W1–W10). For each waffle, 15 g of batter was placed in the center of a Lifetec waffle iron (16.5 cm diameter, 1200 W) and baked for approximately 2 min. After cooling, the wafers were vacuum-packed and stored at room temperature until analysis.

### 4.4. The Semi-Consumer Evaluation

The semi-consumer evaluation was conducted with the participation of a research team consisting of 40 employees and students of the University of Life Sciences in Lublin (22 females and 18 males, 21–52 years). Before evaluation, the panel received basic orientation and training regarding the use of the hedonic scale and sample assessment procedures. Samples were presented in a randomized order and coded with three-digit numbers. The qualitative characteristics assessed included: general appearance, surface, color, taste, aroma, consistency (crispness), and consistency (hardness). A reporting sheet was prepared, on which the evaluators were asked to enter the appropriate scores in the appropriate spaces in the table [29]. The wafers were assessed for quality using a five-point hedonistic scale: 1—poor quality, 2—insufficient quality, 3—sufficient quality, 4—good quality, 5—very good quality.

### 4.5. Preparation of Extracts and Hydrolysates

#### 4.5.1. Preparations of Ethanol Extracts

Ethanol extracts were prepared by weighing 5 g of each wafer type, grinding the sample in a mortar, and adding 15 mL of 50% ethanol. Samples were shaken for 30 min under refrigerated conditions and then centrifuged at 9000× *g* for 10 min at 4 °C. The extraction was repeated three times. Supernatants were decanted, filtered, combined, and adjusted to a final volume of 50 mL with the extraction solvent.

#### 4.5.2. Preparations of PBS Extracts

PBS extracts were prepared by weighing 5 g of each wafer type, grinding the sample in a mortar, and adding 15 mL of phosphate-buffered saline (PBS). Samples were shaken for 30 min under refrigerated conditions and then centrifuged at 9000× *g* for 10 min at 4 °C. The extraction was repeated three times. Supernatants were decanted, filtered, combined, and adjusted to a final volume of 50 mL with PBS.

#### 4.5.3. In Vitro Digestion

Simulated digestion of wafers was performed according to the procedure of Minekus et al. [71] with minor modifications. Briefly, ground wafer samples were subjected to sequential digestion phases simulating the oral, gastric, and intestinal environments. Each phase was carried out under controlled pH, temperature (37 °C), and agitation, using the corresponding digestive enzymes and electrolyte solutions as described in [72].

Oral phase: Ground wafer samples were mixed 1:1 with simulated salivary fluid (SSF) containing the corresponding electrolyte solution and salivary amylase (approximately 75 U/mL). The mixture was adjusted to pH 7 and incubated for 2 min to simulate oral processing.

Gastric phase: The oral bolus was diluted 1:1 with simulated gastric fluid (SGF), including its electrolyte composition, and pepsin (2000 U/mL). Gastric digestion was conducted for 2 h at pH 3.0.

Intestinal phase: After gastric digestion, samples were mixed with simulated intestinal fluid (SIF) containing the appropriate electrolyte solution, bile salts (10 mM), and pancreatic enzymes. Enzymes were added according to INFOGEST recommendations: pancreatin (based on 100 U/mL trypsin activity), bile salts (10 mM). The intestinal phase proceeded for 2 h at pH 7.0.

All electrolyte solutions (SSF, SGF, and SIF) were prepared according to INFOGEST formulations. After digestion, samples were centrifuged (4 °C, 9000× *g*), and supernatants were brought to 50 mL with PBS, frozen, and stored for further analyses.

### 4.6. Peptide Content Determination

The peptide content was determined before and after each digestion stage using the TNBS reagent. L-leucine was used as a standard [73].

### 4.7. Phenolic Content Determination

#### 4.7.1. Total Phenolic Content

A total of 10 μL of the sample was mixed with 40 μL of Folin–Ciocalteu phenol reagent (1:5 in redistilled H_2_O), and after 3 min, 200 μL of 10% Na_2_CO_3_ was added. After 30 min, the absorbance was measured at a wavelength of λ = 725 nm against a reference sample using an Epoch 2 BioTek plate-cell spectrophotometer (BioTek, Winooski, VT, USA). The amount of total phenolic content was calculated in the gallic acid equivalent (GAE) as the mean ± standard deviation (SD) of three replicates [74].

#### 4.7.2. Flavonoid Content

The Lamaison method was used to determine flavonoid content [75]. A 50 μL aliquot of the test sample was transferred to microtiter plates in triplicate, followed by the addition of 50 μL of 2% AlCl_3_ and incubation for 10 min. The resulting reaction mixture was incubated, and absorbance was subsequently measured at λ = 430 nm using an Epoch 2 spectrophotometer (BioTek, Winooski, VT, USA). For the control, the extracts were substituted with the corresponding solvent. Flavonoid content was quantified and expressed as quercetin equivalents (QE = mg Q/g). All measurements were conducted in triplicate to ensure reproducibility.

#### 4.7.3. Phenolic Acid Content

The Arnov method was used for the determination of phenolic acids [76]. 300 µL of distilled H_2_O, 50 µL of the tested extract, 50 µL of 0.5% HCl, 50 µL of Arnov’s reagent and 50 µL of 1 M NaOH were poured into the well of the plates. The resulting reaction mixture was incubated, and absorbance was subsequently measured at λ = 490 nm using an Epoch 2 spectrophotometer (BioTek, Winooski, VT, USA). For the control, the extracts were substituted with the corresponding solvent. Flavonoid content was quantified and expressed as caffeic acid equivalent (CAE = mg CA/g). All measurements were conducted in triplicate.

### 4.8. Qualitative Analysis by LC-QTOF-MS

Qualitative analysis was performed using an LC-QTOF-MS system [35] consisting of an Agilent 1290 Infinity Series liquid chromatograph coupled with an Agilent 6530 Q-TOF LC/MS mass spectrometer (Agilent Technologies, Palo Alto, CA, USA). Chromatographic separation was carried out on a ZORBAX Eclipse Plus C18 column (2.1 × 50 mm, 1.8 µm particle size, Agilent Technologies, Santa Clara, CA, USA). The mobile phase consisted of 0.1% formic acid in acetonitrile (A) and 0.1% formic acid in water (B), a solvent gradient of 5–95% acetonitrile. The flow rate was 0.4 mL/min, and the injection volume was 5 µL. Mass spectra were acquired in the range of 50–1000 Da under both positive (ESI^+^) and negative (ESI^−^) electrospray ionization modes. Data acquisition and processing were performed using MassHunter Acquisition and MassHunter Qualitative Analysis software (version B.10.00, Agilent Technologies, Inc., Santa Clara, CA, USA). Direct database and library searches were conducted using MassHunter Personal Compound Database and Library (PCDL, version B.08.00) with the Find Compounds by Formula (FBF) algorithm. Compound identification was based on the interpretation of MS and MS/MS spectra, including the analysis of characteristic fragment ions. Identification criteria included matching theoretical isotopic patterns with experimental data, evaluating accurate ion masses, and considering potential adduct ions. The quality of the match was expressed as a score value. Compounds with a score ≥ 85 and a mass error ≤ 10 ppm were classified as tentatively identified. Those with a score < 85 or mass deviation > 10 ppm were considered insufficiently identified and were excluded from further qualitative analysis.

### 4.9. The Antioxidant Activity

#### 4.9.1. Determination of the DPPH Radicals Neutralization Capacity

The assay was performed using the method of Brand-Williams et al. (1995) [77]. To 10 µL of the test samples, 250 µL of a 0.06 mM DPPH methanolic solution was added. Absorbance was measured on a plate-cuvette spectrophotometer at a wavelength of λ = 515 nm. The assay was performed in triplicate for each test sample.

Antiradical activity was calculated according to the following formula:

% = [1 − A_s_/A_c_] × 100% (1)

where

%—antiradical activity;
A_s_—absorbance of the tested sample;
A_c_—absorbance of the control sample.

#### 4.9.2. Determination of the ABTS Radical Neutralization Capacity

The assay was performed using the method of Re et al. [78]. Briefly, 10 μL of the test sample was added to the wells of a 96-well plate, followed by the addition of 250 μL of ABTS. Immediately after mixing, absorbance changes over 5 min were measured (measurement every 1 min) using a BioTek Epoch 2 spectrophotometer (Winooski, VT, USA) at a wavelength of 734 nm against a pure solvent standard. The measurement was performed in triplicate.

Antiradical activity was calculated according to the following formula:

% = [1 − A_s_/A_c_] × 100% (2)

where

%—antiradical activity;
A_s_—absorbance of the tested sample;
A_c_—absorbance of the control sample.

#### 4.9.3. The Iron Ion-Chelating Capacity Assay

The method of Decker and Welch (1990) using ferrozine was used to perform the iron ion-chelating capacity assay [79]. Ethanol extracts, PBS, and samples after in vitro digestion were used for the assay. Microtiter plates were loaded with 100 µL of the appropriately diluted sample and 2 µL of 2 mM FeCl_2_. After a 2 min incubation, 4 µL of 5 mM ferrozine was added. The next step was to measure the absorbance after 10 min of reaction on a cuvette plate spectrophotometer at a wavelength of λ = 562 nm against a control sample.

The percentage of Fe^2+^-chelating capacity was calculated according to the following formula:

% = (1 − A_s_/A_c_) × 100 (3)

where

%—chelating capacity;
A_s_—absorbance of the tested sample;
A_c_—absorbance of the control sample.

#### 4.9.4. The Reduction Power Determination

To 50 μL of the extract, we added 50 μL of 200 mM sodium buffer, pH 6.6, followed by 50 μL of 1% potassium ferricyanide. After mixing the reagents, the samples were incubated for 20 min at 50 °C. After incubation, 50 μL of 10% trichloroacetic acid (TCA) was added. The samples were centrifuged (10 min, 4000 rpm). 100 μL of supernatant was then removed and placed in microtiter plates, and 100 μL of deionized water and 20 μL of 0.1% FeCl_3_ were added. The absorbance measurement was performed on a plate-cuvette spectrophotometer at a wavelength of λ = 700 nm against a reference sample in which the extracts were replaced by an appropriate solution, while the remaining reagents remained the same [80].

The antioxidant capacity was determined using a standard curve prepared with Trolox (6-hydroxy-2,5,7,8-tetramethylchroman-2-carboxylic acid), and the results are expressed as µg Trolox/mL for the ABTS, DPPH, and reducing power assays. For the iron chelation assay, a standard curve was prepared using EDTA (disodium edetate), and the results are expressed as mg EDTA/mL.

### 4.10. Enzyme Inhibitory Assays

The inhibitory activities of lipoxygenase (LOX), cyclooxygenase-2 (COX-2), angiotensin I-converting enzyme (ACE), and pancreatic lipase were evaluated to assess the potential anti-inflammatory, antihypertensive, and anti-obesity properties of the samples.

#### 4.10.1. Potential Antihypertension Properties

Angiotensin-converting enzyme (ACE) inhibitory activity was determined following the method of Jakubczyk et al. [13], with slight modification by Karaś et al. [81], which involved scaling down and adapting it to a microplate format.

ACE inhibitory activity was measured using 5 mM HHL as the substrate, with the reaction carried out for 60 min at 37 °C, and the OPA–His–Leu chromophore was detected spectrophotometrically at 390 nm. The inhibition was expressed as EC_50_, representing the sample concentration (mg/mL) required to reduce ACE activity by 50%. EC_50_ values were determined graphically by plotting the percentage inhibition against sample concentrations.

#### 4.10.2. Potential of Anti-Obesity Activity

Lipase inhibitory activity was determined with the method described by Jakubczyk et al. [13]. The reaction mixture containing lipase (100 mg/mL), sample extract, and potassium phosphate buffer (100 mM, pH 7.5) was equilibrated at 37 °C, after which the reaction was initiated by adding p-nitrophenyl acetate (100 mM pNPA). Following a defined incubation period, the formation of p-nitrophenol was monitored by measuring absorbance at 405 nm using a BioTek microplate reader.

Enzyme inhibition was calculated relative to the control sample (enzyme without extract). EC_50_ values were obtained by fitting concentration–response curves based on the percentage inhibition observed at different sample concentrations, and were defined as the concentration required to inhibit 50% of lipase activity.

#### 4.10.3. Potential Anti-Inflammatory Properties

The ability to inhibit lipoxygenase (LOX) activity was determined using spectrophotometric methods described by Szymanowska et al. [82], and the extract concentration (mg/mL) required to achieve 50% inhibition (EC_50_) was determined by plotting the percentage of inhibition against the sample concentration. A 2.5 mM linoleic acid solution in M/15 sodium-potassium buffer, pH 7.0, with Tween 20 detergent added, was used as the substrate. Absorbance was measured after 3 min of incubation of the enzyme solution (soybean lipoxygenase) in phosphate buffer, pH 7.0, with various extract concentrations. The reaction was initiated by the addition of the substrate. Activity was determined kinetically at a wavelength of 234 nm, and a 0.001 mM increase in absorbance per minute was assumed as a unit of lipoxygenase activity. 100% enzyme activity was determined similarly without the addition of extract.

Cyclooxygenase-2 (COX-2) inhibition was determined according to the manufacturer’s procedure, using oxidation of TMPD (N,N,N′,N′-tetramethyl-p-phenylenediamine) as a chromogenic substrate with the Cayman Chemical COX Colorimetric Inhibitor Screening Assay Kit (Ann Arbor, MI, USA). Results are expressed as EC_50_—the concentration (mg/mL) causing 50% enzyme inhibition. Changes in absorbance were measured after 5 min of incubation of the enzyme with various extract concentrations. The reaction was initiated by the addition of TMPD and arachidonic acid solution and kinetically measured for 2 min against a blank containing buffer only at a wavelength of 590 nm. Percent inhibition was determined relative to enzyme activity without the addition of extract.

### 4.11. Cancer Cell Viability Assay

#### 4.11.1. Cell Culture

Cell lines of human gastric adenocarcinoma AGS (ECACC No. 89090402) (Sigma-Aldrich, Poznań, Poland), and human colorectal adenocarcinoma HT-29 (ATCC HTB-38) (local distributor of ATCC—LGC Standards, Łomianki, Poland) were grown in DMEM-F12 medium containing 10% FBS (Fetal bovine serum) and 1% of antibiotic-antimycotic solution in humidified atmosphere of 5% CO_2_ at 37 °C.

#### 4.11.2. Viability Assay

The potential antiproliferative activity of wafer samples obtained after in vitro digestion was analyzed with WST-1 Assay Protocol for Cell Viability (Abcam, Cambridge, UK). The cells were seeded into a 96-well plate at an initial density of 2 × 10^4^ cells/well and after 24 h the culture medium was exchanged with fresh medium (control) or replaced with a digest extracts dissolved in culture medium to reach the final concentrations of 5, 2.5, 1.25, 0.675 and 0.3125 mg/mL of culture medium) and the cells were cultured for the next 24 h. Absorbance at 440 nm and 650 nm (as background) was evaluated using the Epoch 2 spectrophotometer by BioTek (Winooski, VT, USA).

### 4.12. Statistical Analysis

Experimental data are presented as the mean S.D. of data obtained from three independent samples of each extract. Statistical analysis was performed using STATISTICA 13.3 to compare means using Tukey’s test at a 95% confidence level (*p* < 0.05).

## 5. Conclusions

The addition of almond peel to the wafers influenced their appearance, crispness, and color, making them darker and progressively crispier with increasing levels of the additive. Wafers containing almond peel proved to be a good source of bioactive compounds, whose extractable content depended largely on the applied extraction system. The in vitro digestion process promoted the release of these compounds, enhancing the antioxidant, antihypertensive, and anti-obesity potential of the wafers. It also increased the susceptibility of wafer proteins to enzymatic hydrolysis compared with the control, as shown by the rising number of peptides detected at each stage of digestion, which supports the potential of almond peel as a functional food ingredient. From a functional food perspective, the decrease in EC_50_ values after digestion is particularly meaningful and affirms that almond skin contributes to stronger antioxidant and enzyme inhibitory activities, suggesting possible health-promoting effects in vivo in areas such as cardiovascular protection and obesity management.

Nonetheless, the study presents several limitations. The findings are derived primarily from in vitro experiments; the observed anticancer activity is modest, and high levels of almond peel may pose challenges for sensory acceptance, which is a key factor in consumer acceptance of functional foods. The absence of a clear dose–response relationship at lower supplementation levels further illustrates the complexity of interactions among components of the food matrix, especially between proteins, peptides, and phenolic compounds.

Future research should therefore extend beyond the addition-based assessment of activity and take a more comprehensive approach. Promising directions include animal studies to confirm antihypertensive effects, process optimization to balance bioactive functionality with desirable sensory attributes, and human intervention trials to verify actual health benefits and to identify the specific compounds responsible for the inhibitory activities observed in vitro.

## Figures and Tables

**Table 1:** LC-QTOF-MS analysis.

	PBS		EtOH		TRW	
	WK	W10	WK	W10	WK	W10
Phenolic acids						
Caffeic acid					+	+
Ferulic acid					+	+
p-Coumaric acid					+	+
Fatty acids and amino acids						
Linoleic acid				+	+	+
Methionine					+	+
Tryptophan	+	+	+	+	+	+
Valine	+	+	+	+	+	+
Phenylalanine	+	+	+	+	+	+
Tyrosine	+	+	+		+	+
Arginine		+	+	+	+	+
Leucine					+	+
Isoleucine	+	+	+	+	+	+
Vitamins						
Vitamin B2 (riboflavin)		+				
Vitamin B3 (niacin)			+			
Vitamin B5 (pantothenic acid)	+	+	+	+	+	+
Vitamin E (γ tocopherol)				+		+
Flavonoids and other polyphenols						
Kaempferol				+		
Apigenin7-O-apiosyl-glucoside	+	+	+	+		
Isorhamnetin-3-O-rutinoside		+				
Isorhamnetin-3-O-glucoside				+		
Catechin				+		
Epicatechin				+		
Naringenin				+		
Eriodictyol				+		
Secoisolariciresinol				+		
Piceatannol 3-O-glucoside				+		+
Benzaldehyde					+	+
Mandelic acid						+

+ indicates presence.

**Table 2:** Identification of active compounds using the LC-QTOF-MS technique.

	Molecular Formula	R_t_ (min)	Observed Mass	*m*/*z*	Detected Ion	Score	Dif (ppm)
Phenolic acids							
Caffeic acid	C_9_H_8_O_4_	1.953	180.0423	239.0575	[M+CH_3_COO]^−^	75.3	8.53
Ferulic acid	C_10_H_10_O_4_	1.953	194.0579	239.0575	[M+HCOO]^−^	75.3	7.92
p-Coumaric acid	C_9_H_8_O_3_	0.582	164.0473	165.0539	[M+H]^+^	76.93	−2.10
Fatty acids and amino acids							
Linoleic acid	C_18_H_32_O_2_	14.734	280.2402	281.2469	[M+H]^+^	78.83	−0.20
Methionine	C_5_H_11_NO_2_S	0.549	149.0510	150.0591	[M+H]^+^	96.19	5.44
Tryptophan	C_11_H_12_N_2_O_2_	1.914	204.0899	205.0979	[M+H]^+^	97.27	3.30
Valine	C_5_H_11_NO_2_	9.157	117.0792	118.0865	[M+H]^+^	95.93	1.97
Phenylalanine	C_9_H_11_NO_2_	0.932	165.079	166.0861	[M+H]^+^	99.8	−1.01
Tyrosine	C_9_H_11_NO_3_	0.582	181.0739	182.0811	[M+H]^+^	99.84	−0.17
Arginine	C_6_H_14_N_4_O_2_	0.383	174.1117	175.1184	[M+H]^+^	98.42	−3.27
Leucine	C_6_H_13_NO_2_	1.165	131.0946	132.1013	[M+H]^+^	97.42	−4.09
Isoleucine	C_6_H_13_NO_2_	0.616	131.0947	132.1020	[M+H]^+^	99.56	0.72
Vitamins							
Vitamin B2 (riboflavin)	C_17_H_20_N_4_O_6_	4.295	376.1383	377.1446	[M+H]^+^	92.76	−1.26
Vitamin B3 (niacin)	C_6_H_5_NO_2_	0.489	123.0325	124.0398	[M+H]^+^	77.74	3.90
Vitamin B5 (pantothenic acid)	C_9_H_17_NO_5_	1.215	219.1107	220.1181	[M+H]^+^	99.19	0.89
Vitamin E (γ tocopherol)	C_28_H_48_O_2_	15.932	416.3654	461.3636	[M+HCOO]^−^	84.9	−3.22
Flavonoids and other polyphenols							
Kaempferol	C_15_H_10_O_6_	6.992	286.0477	287.0534	[M+H]^+^	81.77	−5.36
Apigenin7-O-apiosyl-glucoside	C_26_H_28_O_14_	4.330	564.1488	565.1558	[M+H]^+^	84.21	2.10
Isorhamnetin-3-O-rutinoside	C_28_H_32_O_16_	5.170	624.169	625.1766	[M+H]^+^	99.74	0.50
Isorhamnetin-3-O-glucoside	C_22_H_22_O_12_	5.270	478.1111	479.1182	[M+H]^+^	91.68	0.75
Catechin	C_15_H_14_O_6_	4.278	290.079	291.0872	[M+H]^+^	76.23	−0.29
Epicatechin	C_15_H_14_O_6_	3.662	290.079	291.0874	[M+H]^+^	75.5	2.33
Naringenin	C_15_H_12_O_5_	6.826	272.0685	273.0765	[M+H]^+^	78	1.47
Eriodictyol	C_15_H_12_O_6_	5.610	288.0634	289.0720	[M+H]^+^	80.94	4.81
Secoisolariciresinol	C_20_H_26_O_6_	8.889	362.1729	361.1660	[M-H]^−^	92.5	−4.34
Piceatannol 3-O-glucoside	C_20_H_20_O_8_	0.399	406.1264	407.1327	[M+H]^+^	88.1	−0.30
Benzaldehyde	C_7_H_6_O	0.932	106.0419	107.0490	[M+H]^+^	81.74	−0.08
Mandelic acid	C_8_H_8_O_3_	3.563	152.0473	153.0558	[M+H]^+^	78.18	8.04

**Table 3:** The effect of wafers on enzyme activity.

		Sample				
		WK	W1	W2	W5	W10
ACE EC_50_ [mg/mL]	PBS EtOH TRW	9.680 ± 1.065 ^aA^ 3.859 ± 0.367 ^aB^ 0.280 ± 0.013 ^aC^	6.137 ± 1.04 ^bA^ 2.160 ± 0.162 ^bB^ 0.268 ± 0.020 ^aC^	4.239 ± 0.210 ^cA^ 0.907 ± 0.050 ^cB^ 0.254 ± 0.014 ^abC^	4.064 ± 0.244 ^cA^ 0.879 ± 0.057 ^cB^ 0.239 ± 0.015 ^bC^	3.615 ± 0.398 ^cA^ 0.803 ± 0.100 ^cB^ 0.226 ± 0.027 ^bC^
Lipase EC_50_ [mg/mL]	PBS EtOH TRW	12.711 ± 1.398 ^aA^ 4.388 ± 0.219 ^aB^ 1.780 ± 0.178 ^aC^	8.571 ± 0.717 ^bA^ 3.846 ± 0.153 ^bB^ 0.586 ± 0.053 ^bC^	7.227 ± 0.578 ^cA^ 3.452 ± 0.138 ^cB^ 0.310 ± 0.019 ^cC^	7.169 ± 0.476 ^dA^ 3.100 ± 0.123 ^dB^ 0.247 ± 0.022 ^cC^	5.948 ± 0.686 ^dA^ 3.014 ± 0.180 ^dB^ 0.232 ± 0.024 ^cC^
LOX EC_50_ [mg/mL]	PBS EtOH TRW	nd. nd. 0.288 ± 0.011 ^a^	nd. 2.655 ± 0.273 ^aA^ 0.273 ± 0.009 ^aB^	nd. 2.673 ± 0.299 ^aA^ 0.267 ± 0.0089 ^aB^	nd. 2.762 ± 0.428 ^aA^ 0.265 ± 0.013 ^aB^	nd. 2.042 ± 0.397 ^bA^ 0.234 ± 0.008 ^bB^
COX-2 EC_50_ [mg/mL]	PBS EtOH TRW	nd. 2.639 ± 0.157 ^aA^ 1.154 ± 0.111 ^aB^	nd. 2.029 ± 0.107 ^bA^ 0.720 ± 0.062 ^bB^	nd. 2.029 ± 0.141 ^bA^ 0.461 ± 0.026 ^cB^	nd. 1.649 ± 0.079 ^cA^ 0.411 ± 0.014 ^cB^	nd. 1.388 ± 0.018 ^dA^ 0.339 ± 0.011 ^dB^

PBS—buffer extract; EtOH—ethanol extract; TRW—extract after in vitro digestion. WK—control wafers, W1–W10—wafers with almond peels addition (from 1% to 10% flour substitution, respectively); nd.—not detected. All values are mean ± standard deviation for triplicate experiments. Different capital letters indicate statistically significant differences within the same sample, but between the different types of extracts (*p* < 0.05). Values denoted by different small letters indicate statistically significant differences within the rows (*p* < 0.05).

## Data Availability

All of the data are available in the article.

## Supplementary Materials

The following supporting information can be downloaded at: https://www.mdpi.com/article/10.3390/molecules31010129/s1, Figure S1. Exemplary calibration curves for determining EC50 values for enzyme inhibition: (A) ACE, (B) lipase, (C) LOX, (D) COX-2. Table S1. Matrix Correlation Coefficients (*p* < 0.05).
