# Supplementary material for: The Health-Promoting Potential of Wafers Enriched with Almond Peel
Source: Molecules. 2025 Dec 30;31(1):129. doi: 10.3390/molecules31010129 (PMC12788093; doi:10.3390/molecules31010129)
Supplement: Supplementary file 1 [file molecules-31-00129-s001.zip › molecules-3990028-supplementary.pdf]

Supplementary material: The health-promoting potential of wafers enriched with almond peel

Urszula Szymanowska, Monika Karaś, Ivo Oliveira, Sílvia Afonso, Barbara Chilczuk and Katarzyna Lisiecka

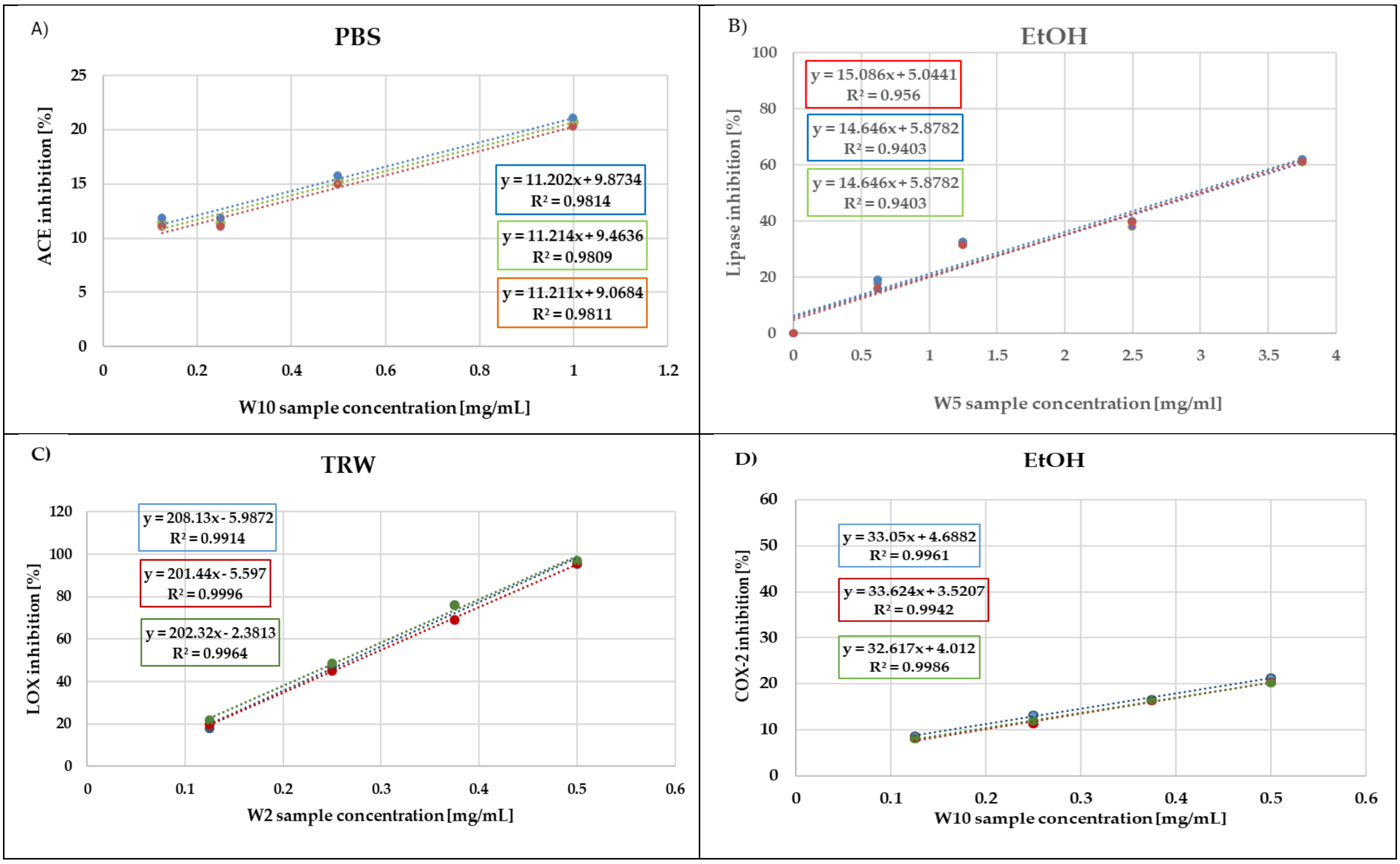

Fig. S1. Exemplary calibration curves for determining EC_{50} values for enzyme inhibition: A) ACE, B) lipase, C) LOX, D) COX-2

Table S1. Matrix Correlation Coefficients (*P*<0.05)

	TP-PBS	TP-EtOH	TP-D	F-PBS	F-EtOH	F-D	PA-PBS	PA-EtOH	PA-D
RP-PBS	.9055<br>p=.034	.9303<br>p=.022	.9260<br>p=.024	.9135<br>p=.030	.9375<br>p=.019	.9586<br>p=.010	.8875<br>p=.045	.8392<br>p=.075	.9068<br>p=.034
RP-EtOH	.9152<br>p=.029	.9439<br>p=.016	.9151<br>p=.029	.9393<br>p=.018	.9058<br>p=.034	.9279<br>p=.023	.8635<br>p=.059	.8513<br>p=.067	.9097<br>p=.032
RP-D	.8395<br>p=.075	.8787<br>p=.050	.8599<br>p=.062	.8697<br>p=.055	.8615<br>p=.061	.8921<br>p=.042	.8087<br>p=.098	.7575<br>p=.138	.8365<br>p=.077
Ch-PBS	.9697<br>p=.006	.9897<br>p=.001	.9508<br>p=.013	.9702<br>p=.006	.9442<br>p=.016	.9566<br>p=.011	.9027<br>p=.036	.9244<br>p=.025	.9725<br>p=.005
Ch-EtOH	.9467<br>p=.015	.9707<br>p=.006	.9382<br>p=.018	.9607<br>p=.009	.9294<br>p=.022	.9469<br>p=.015	.8883<br>p=.044	.8917<br>p=.042	.9442<br>p=.016
Ch-D	.9873<br>p=.002	.9718<br>p=.006	.9957<br>p=.000	.9755<br>p=.005	.9999<br>p=.000	.9969<br>p=.000	.9832<br>p=.003	.9692<br>p=.006	.9783<br>p=.004
ABTS-PBS	.9819<br>p=.003	.9636<br>p=.008	.9947<br>p=.000	.9928<br>p=.001	.9849<br>p=.002	.9836<br>p=.003	.9809<br>p=.003	.9626<br>p=.009	.9590<br>p=.010
ABTS-EtOH	.8765<br>p=.051	.9071<br>p=.033	.9011<br>p=.037	.8802<br>p=.049	.9200<br>p=.027	.9440<br>p=.016	.8617<br>p=.060	.8037<br>p=.101	.8834<br>p=.047
ABTS-D	.9882<br>p=.002	.9755<br>p=.005	.9617<br>p=.009	.9654<br>p=.008	.9554<br>p=.011	.9477<br>p=.014	.9383<br>p=.018	.9833<br>p=.003	.9870<br>p=.002
DPPH-PBS	.8414<br>p=.074	.8624<br>p=.060	.8583<br>p=.063	.8939<br>p=.041	.8365<br>p=.077	.8598<br>p=.062	.8138<br>p=.094	.7778<br>p=.121	.8167<br>p=.092
DPPH-EtOH	.8119<br>p=.095	.8571<br>p=.063	.8316<br>p=.081	.8458<br>p=.071	.8324<br>p=.080	.8663<br>p=.057	.7758<br>p=.123	.7240<br>p=.167	.8101<br>p=.096
DPPH-D	.9858<br>p=.002	.9688<br>p=.007	.9706<br>p=.006	.9525<br>p=.012	.9761<br>p=.004	.9665<br>p=.007	.9561<br>p=.011	.9820<br>p=.003	.9881<br>p=.002
ACE-PBS	-.9712<br>p=.006	-.9362<br>p=.019	-.9580<br>p=.010	-.9375<br>p=.019	-.9587<br>p=.010	-.9402<br>p=.017	-.9571<br>p=.011	-.9862<br>p=.002	-.9645<br>p=.008
ACE-EtOH	-.9634<br>p=.008	-.9343<br>p=.020	-.9396<br>p=.018	-.9238<br>p=.025	-.9406<br>p=.017	-.9229<br>p=.025	-.9327<br>p=.021	-.9777<br>p=.004	-.9632<br>p=.008
ACE-D	-.9691<br>p=.006	-.9851<br>p=.002	-.9644<br>p=.008	-.9746<br>p=.005	-.9607<br>p=.009	-.9737<br>p=.005	-.9226<br>p=.026	-.9213<br>p=.026	-.9670<br>p=.007
L-PBS	-.9661<br>p=.007	-.9261<br>p=.024	-.9696<br>p=.006	-.9369<br>p=.019	-.9746<br>p=.005	-.9564<br>p=.011	-.9769<br>p=.004	-.9792<br>p=.004	-.9543<br>p=.012
L-EtOH	-.9973<br>p=.000	-.9849<br>p=.002	-.9829<br>p=.003	-.9912<br>p=.001	-.9719<br>p=.006	-.9680<br>p=.007	-.9594<br>p=.010	-.9846<br>p=.002	-.9868<br>p=.002
L-D	-.9276<br>p=.023	-.8645<br>p=.059	-.9257<br>p=.024	-.8980<br>p=.038	-.9200<br>p=.027	-.8889<br>p=.044	-.9485<br>p=.014	-.9693<br>p=.006	-.9045<br>p=.035
COX-PBS	--<br>p= ---	--<br>p= ---	--<br>p= ---	--<br>p= ---	--<br>p= ---	--<br>p= ---	--<br>p= ---	--<br>p= ---	--<br>p= ---
COX-EtOH	-.9676<br>p=.007	-.9383<br>p=.018	-.9900<br>p=.001	-.9849<br>p=.002	-.9773<br>p=.004	-.9724<br>p=.005	-.9861<br>p=.002	-.9556<br>p=.011	-.9354<br>p=.019
COX-D	-.9833<br>p=.003	-.9553<br>p=.011	-.9696<br>p=.006	-.9537<br>p=.012	-.9696<br>p=.006	-.9550<br>p=.011	-.9625<br>p=.009	-.9904<br>p=.001	-.9779<br>p=.004
LOX-PBS	--<br>p= ---	--<br>p= ---	--<br>p= ---	--<br>p= ---	--<br>p= ---	--<br>p= ---	--<br>p= ---	--<br>p= ---	--<br>p= ---
LOX-EtOH	.7319<br>p=.160	.6229<br>p=.262	.7324<br>p=.159	.7026<br>p=.186	.7125<br>p=.177	.6574<br>p=.228	.7934<br>p=.109	.8283<br>p=.083	.6864<br>p=.201
LOX-D	-.8748<br>p=.052	-.8946<br>p=.040	-.9064<br>p=.034	-.8709<br>p=.055	-.9305<br>p=.022	-.9492<br>p=.014	-.8784<br>p=.050	-.8114<br>p=.095	-.8803<br>p=.049

Abbreviations in the table: PBS-phosphate buffer, EtOH-enthanol extract, D- extract after in vitro digestion, TP-total phenolic content, F-total flavonoid content, PA- total phenolic acid content, RP- Reducing Power, Ch- Chelating Activity, ABTS- $ABTS^{+}$ radical scavenging, DPPH-DPPH radical scavenging, ACE- Angiotensin-Converting Enzyme inhibition activity, L-Lipase inhibition activity, COX-Cyclooxygenase inhibition activity, LOX-Lipoxygenase inhibition activity.
